# Supplementary material for: An antagonistic epigenetic mechanism regulating gene expression in pollen revealed through single-nucleus multiomics
Source: Nat Commun. 2025 Nov 27;16:10662. doi: 10.1038/s41467-025-65686-z (PMC12660930; doi:10.1038/s41467-025-65686-z)
Supplement: Supplementary file 1 — Supplementary Information [file 41467_2025_65686_MOESM1_ESM.pdf]

Supplementary Information

Supplementary Figures 1-13

Supplemental References #66-67

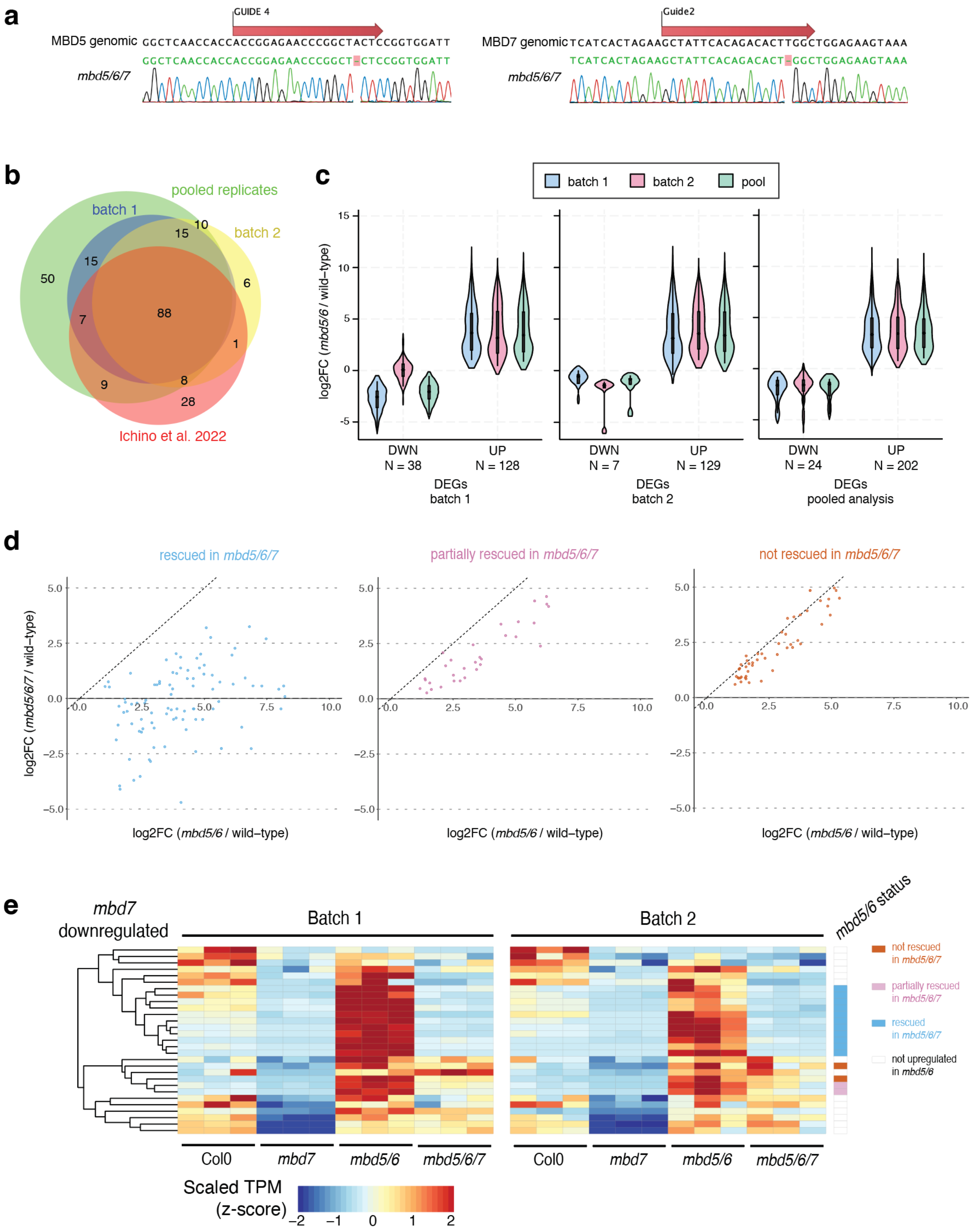

Supplementary Figure 1: pollen RNA-seq analysis related to Fig. 1. A) Sequence of the guides used to generate CRISPR mutants of MBD5 (left) and MBD7 (right), with Sanger sequencing chromatograms of resulting mutant lines. B) Overlap between *mbd5/6* upregulated transcripts in pollen identified in both replicates and pooled analysis from this study, as well as Ichino *et al.* 2022[6] bulk pollen data. C) Comparison of DESeq2[47] $\log_2$(*mbd5/6* / wild-type) expression fold change estimates from batch 1 data, batch 2, and the combined (pooled) analysis, over significantly differentially expressed transcripts identified in batch 1 alone,

batch 2 alone, and the pooled analysis combining both. DWN = downregulated in *mbd5/6* vs. WT, UP = upregulated in *mbd5/6* vs. WT. D) Scatterplot of DESeq2-estimated log2 fold change (log2FC) in *mbd5/6* vs. *mbd5/6/7*, for the 202 loci significantly upregulated in *mbd5/6.* Loci were separated based on rescue status, identified based on hierarchical clustering (see Fig. 1d). E) Heatmap of pollen RNA-seq expression for the 29 transcripts significantly downregulated in *mbd7*. Rightmost column flags *mbd5/6* upregulated transcripts rescued, partially rescued, and not rescued in *mbd5/6/7* (see Fig. 1d). TPM: transcripts per million, scaled by row.

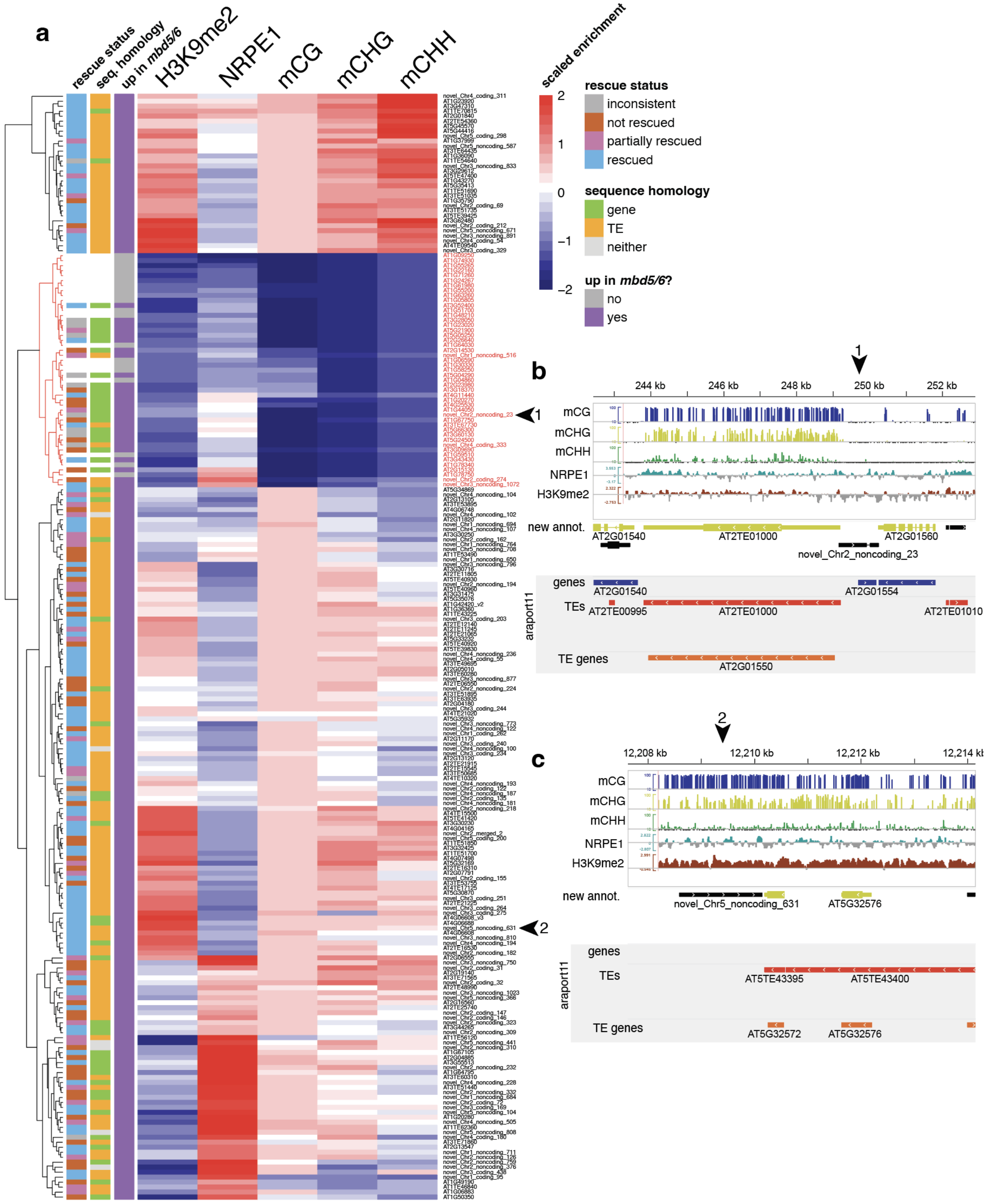

Supplementary Figure 2: Annotation of *mbd5/6* upregulated transcripts. (A) Heatmap of normalized average H3K9me2 signal[55], NRPE1 signal[56], and DNA methylation in the CG, CHG and CHH contexts[25], over all 202 *mbd5/6* upregulated transcripts. As a control, a set of 20 randomly selected control genes is also included (flagged grey in 'up in *mbd5/6*' column). The 'rescue status' column flags loci based on their rescue status in Fig. 1d ('rescued', 'partially rescued', or 'not rescued'; all others flagged as 'inconsistent'). The 'seq. homology' column indicates whether the locus was considered to have higher homology to existing

Araport11[61] gene or TE/TE gene annotations (see methods). Dendogram and transcript IDs highlighted red are putative expressed genes, based on the hierarchical clustering. (B) Example novel annotation that was considered a gene or gene-like (‘novel_Chr2_noncoding_23’). (C) Example novel annotation that was considered a TE, with TE-like methylation and H3K9me2 (‘novel_Chr5_noncoding_631’).

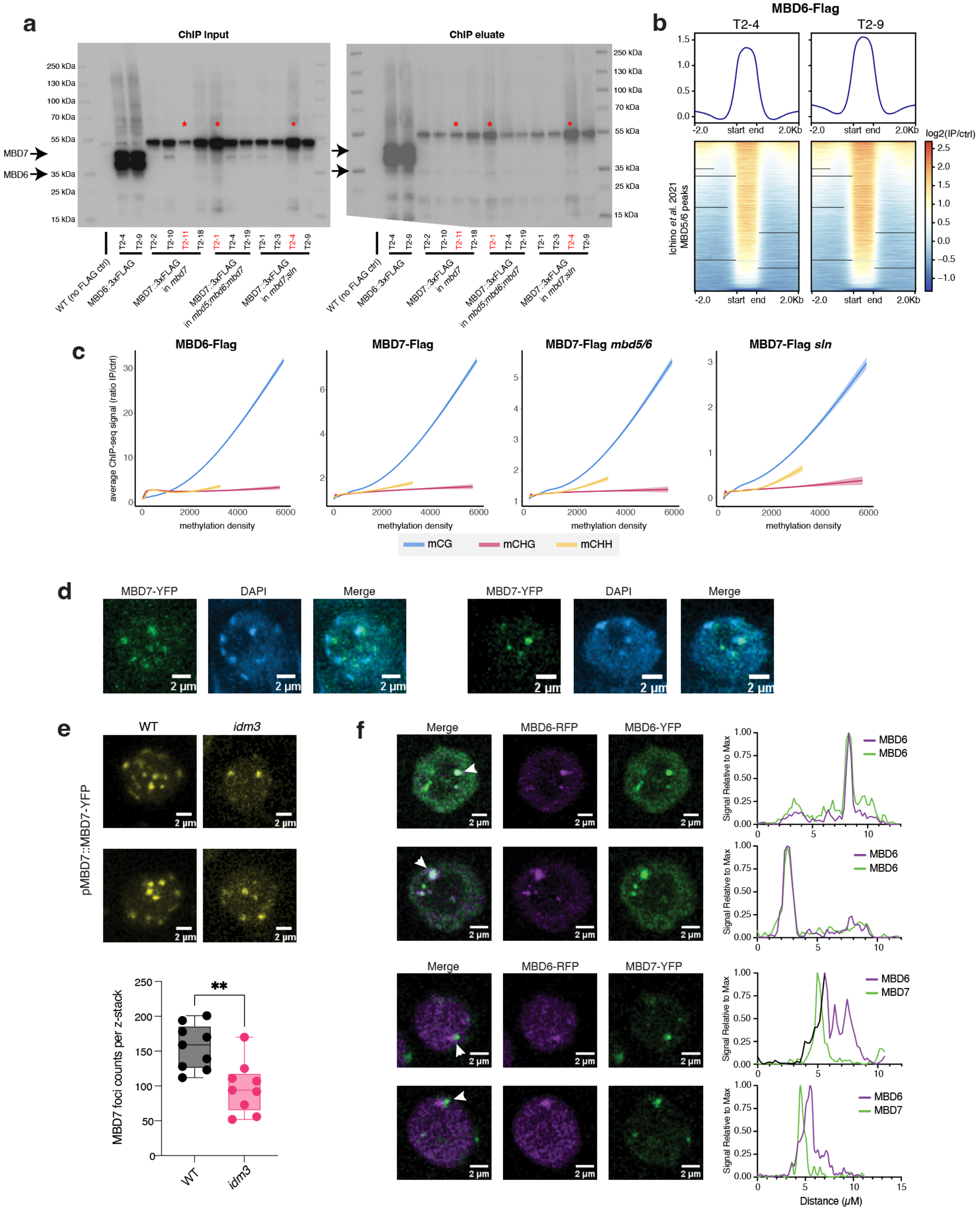

Supplementary Figure 3: MBD7 ChIP-seq validation and immunofluorescence assays related to Fig. 2. A) Western blot of MBD6-Flag and MBD7-Flag, MBD7-Flag *mbd5/6*, and MBD7-Flag *sln* lines used for ChIP-seq. Left is the blot of chromatin input to the ChIP, right is post-IP. Expected sizes are 35 kDa for MBD6-Flag and 45 kDa for MBD7-Flag, although we always detect bands above these sizes for both proteins.

Red stars indicate samples that were censored due to either unusually weak or strong signal in the input relative to the other lines. Each sample was a different independent transgenic line, numbered T2-[#]. B) Plot of MBD6-Flag ChIP-seq signal for both of the transgenic lines used, over MBD5/6 peaks identified in [4]. C) Loess regression and standard deviation comparing ChIP-seq signal in indicated genotype (average of all samples) to density of methylated CG (mCG), CHG (mCHG) or CHH (mCHH) sites (see methods). ChIP-seq signal shown is the ratio over the no-FLAG control and represents average of both lines for each genotype. Methylation data used was two published replicates from inflorescences with unopened flower buds[25]. The two replicates were pooled prior to analysis to increase depth. D) Two representative examples of nuclei expressing pMBD7::MBD7-YFP and incubated with DAPI to stain chromocenters. E) Example 3D reconstructed images of pMBD7::MBD7-YFP (top) from a Z-stack of root cells alongside quantification of number of foci detected across z-stack images per root tip (bottom, one root tip per n = 9 independent transformed lines per genotype) in both wild-type and *ibm1*. ** = p = 0.0025, two-sided unpaired t-test. F) (left) Representative images of nuclei co-expressing pMBD6::MBD6-RFP and either pMBD6::MBD6-YFP (top) or pMBD7::MBD7-YFP (bottom). (right) quantification of RFP and YFP signal strength in nuclei to the left. Signal was measured along a straight line drawn across the indicated foci (white arrow).

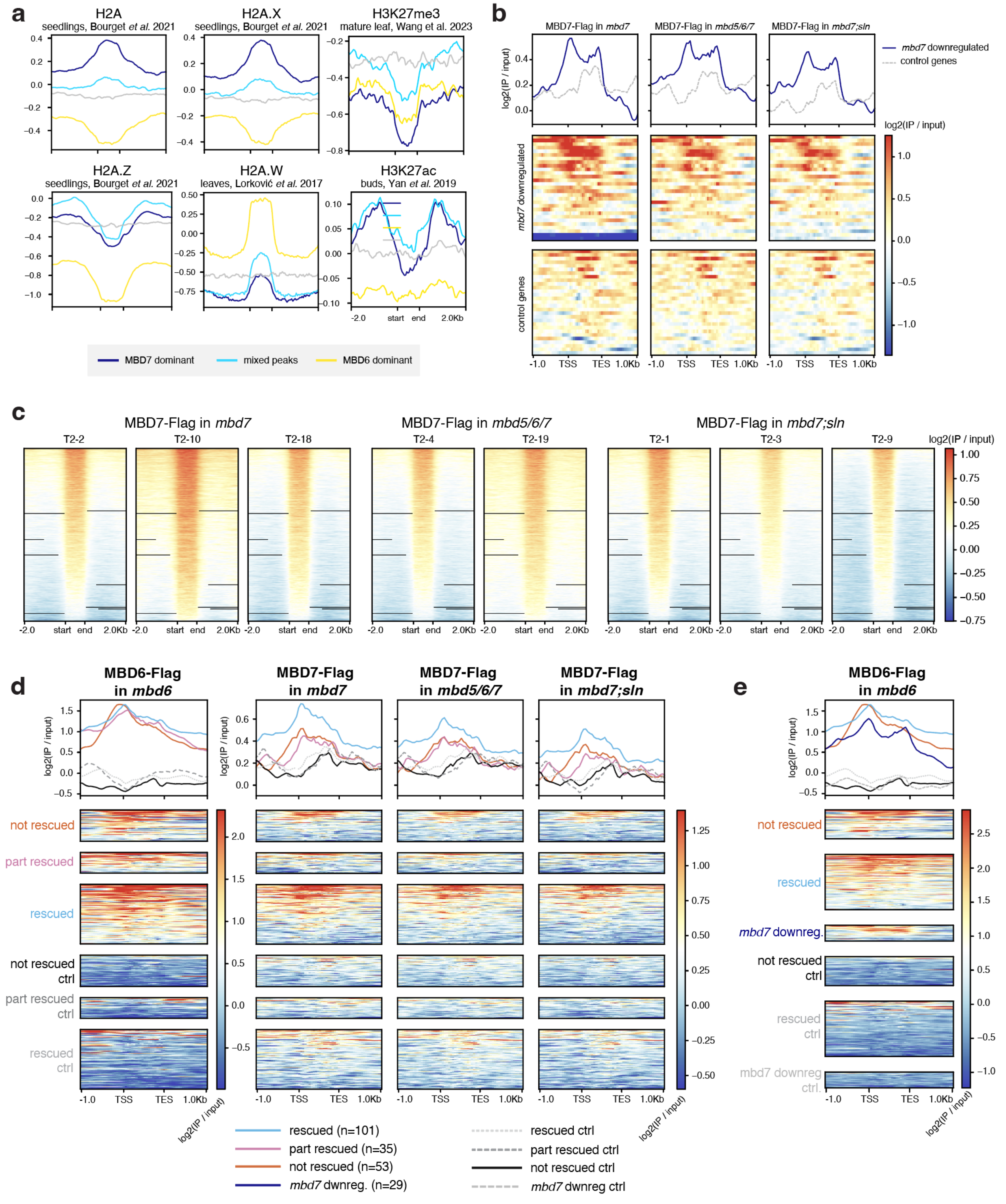

Supplementary Figure 4: MBD7 ChIP-seq analysis related to Fig. 2. A) Metaplots of various epigenetic marks (histone H2A, H2A.X, and H2A.Z[63], H2A.W[66], H3K27me3[64], H3K27ac[67]) over the MBD7-dominant, MBD6-dominant, and mixed peaks from Fig. 2a. Shuffled peaks shown in grey. B) Metaplots and corresponding heatmaps of MBD7-Flag signal (log_2 of IP over no-FLAG control) in indicated background over transcripts downregulated in *mbd7*, and a set of control genes matched for expression and length.

Plotted values represent the average of multiple independent transgenic lines (N=3 for *mbd7*, N=2 for *mbd5/6/7*, and N=3 for *mbd7;sln*). C) Heatmaps of MBD7-Flag signal (log_2 of IP over no-FLAG control), for multiple independent transgenic lines in the indicated mutant background. Heatmaps all share same row order, which was sorted based on the MBD7-Flag *mbd7* T2-2 sample (first column), and is the same as in Fig. 2a. D) Metaplots and corresponding heatmaps of MBD7-Flag signal (log_2 of IP over no-FLAG control) in indicated background over pollen *mbd5/6* upregulated transcripts that were rescued, partially rescued, and not rescued by loss of MBD7 (see clustering in Fig. 1d). Control transcripts are an equal number of randomly selected genes matched for expression level and length. Plotted values represent the average of multiple independent transgenic lines (N=3 for *mbd7*, N=2 for *mbd5/6/7*, and N=3 for *mbd7;sln*). E) Metaplot and heatmap of MBD6-Flag signal (log_2 of IP over no-FLAG control, avg. of 2 transgenic lines) over *mbd5/6* rescued and not rescued transcripts, as well as *mbd7* downregulated transcripts, and their matched control genes. Color legend for metaplot same as in (D).

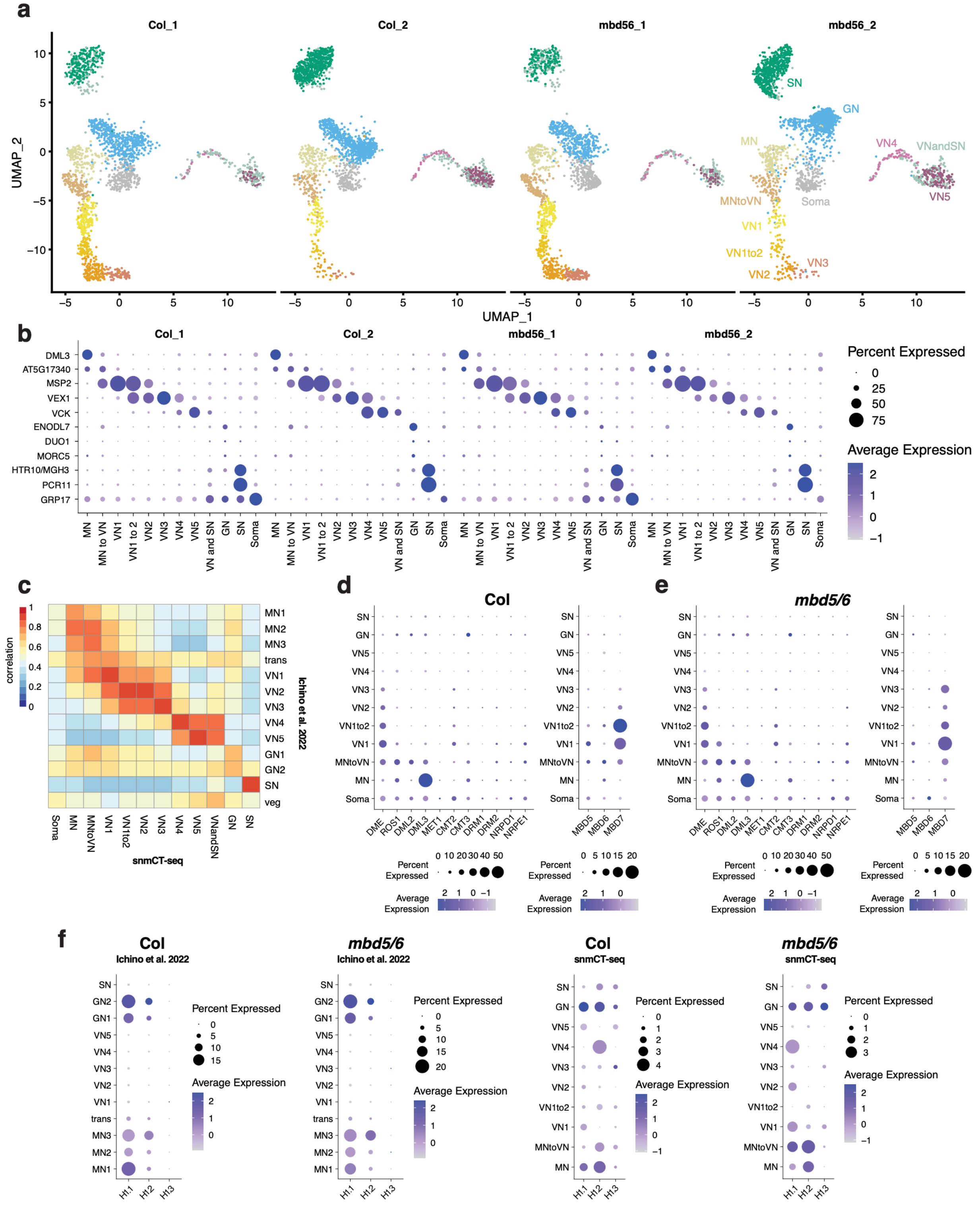

Supplementary Figure 5: snmCT-seq transcriptome data validation related to Fig. 3. (A) UMAP plots of Col and mbd5/6 samples from both independent experiments (expt 1 and expt 2), shown separately. Clusters are labeled on far right plot. Col_1 = Col from expt 1. B) Expression of key marker genes in each cluster, across each experiment and genotype. DML3 and AT5G17340 are elevated in MN. The three VN markers are for early post-mitotic (MSP2), mid-stage (VEX1) and mature (VCK) VN. ENODL7, DUO1 and MORC5 mark GN, HTR10/MGH3 and PCR11 mark SN, and GRP17 marks somatic nuclei. C) Heatmap of

correlation of RNA-seq data between clusters identified in snmCT-seq (this study) and a prior 10x pollen dataset.[6] D) Dotplots of average expression of indicated genes across snmCT-seq clusters in Col. E) Dotplots of average expression of indicated genes across snmCT-seq clusters in *mbd5/6*. F) Dotplots of average expression of the three H1 homologs in both published 10x pollen data[6] and the snmCT-seq data (this study).

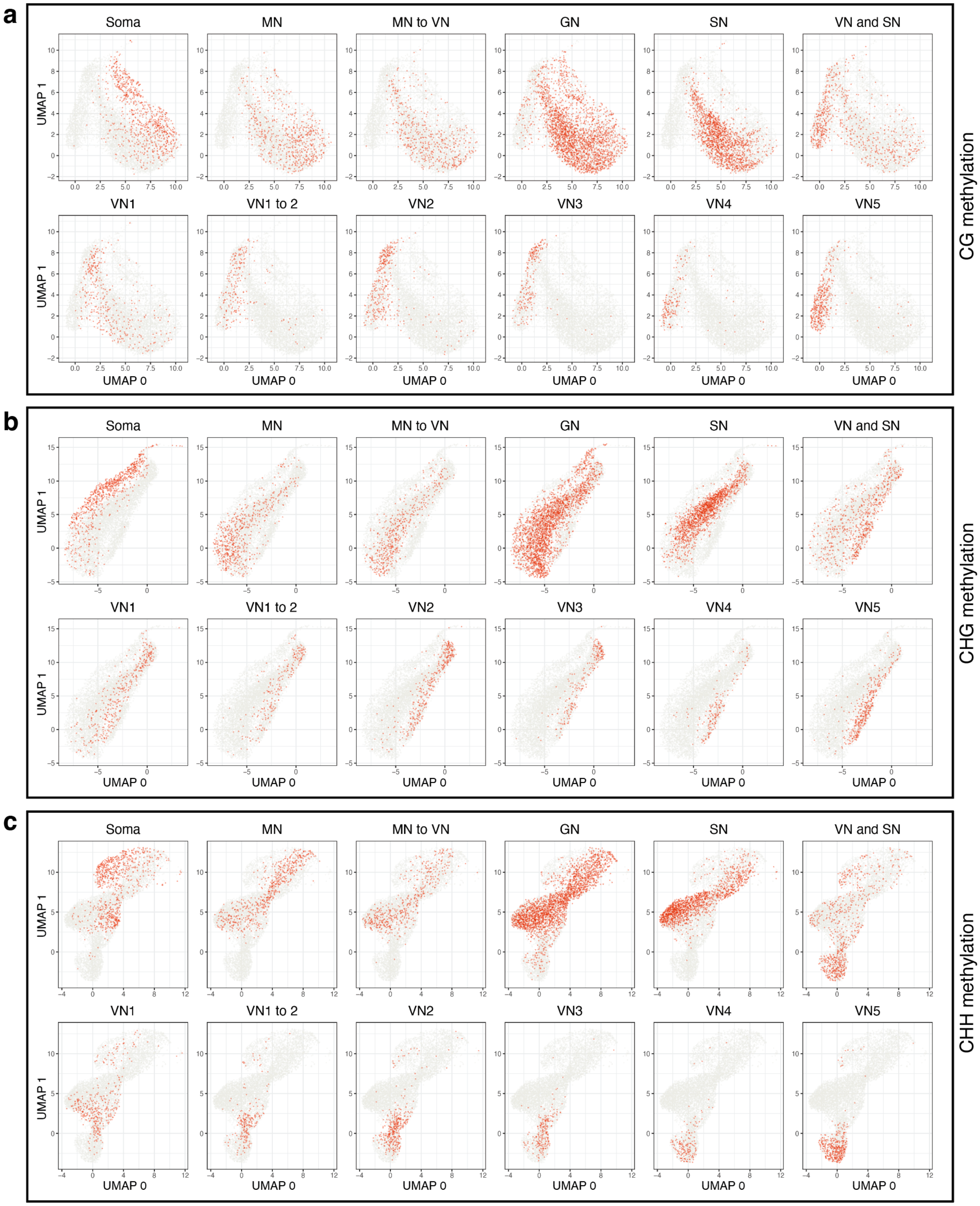

Supplementary Figure 6: unsupervised clustering of nuclei based on snmCT-seq methylation data, related to Fig. 4. (A) UMAP projection of the full snmCT-seq dataset, using the CG methylation data averaged over non-overlapping, 25 kb bins tiled genome-wide. Each plot highlights nuclei in the indicated cluster, using cluster identities based on the snmCT-seq transcriptome data (Fig. 3b). (B) Same as A, but using the CHG methylation data. (C) Same as A, but using the CHH methylation data.

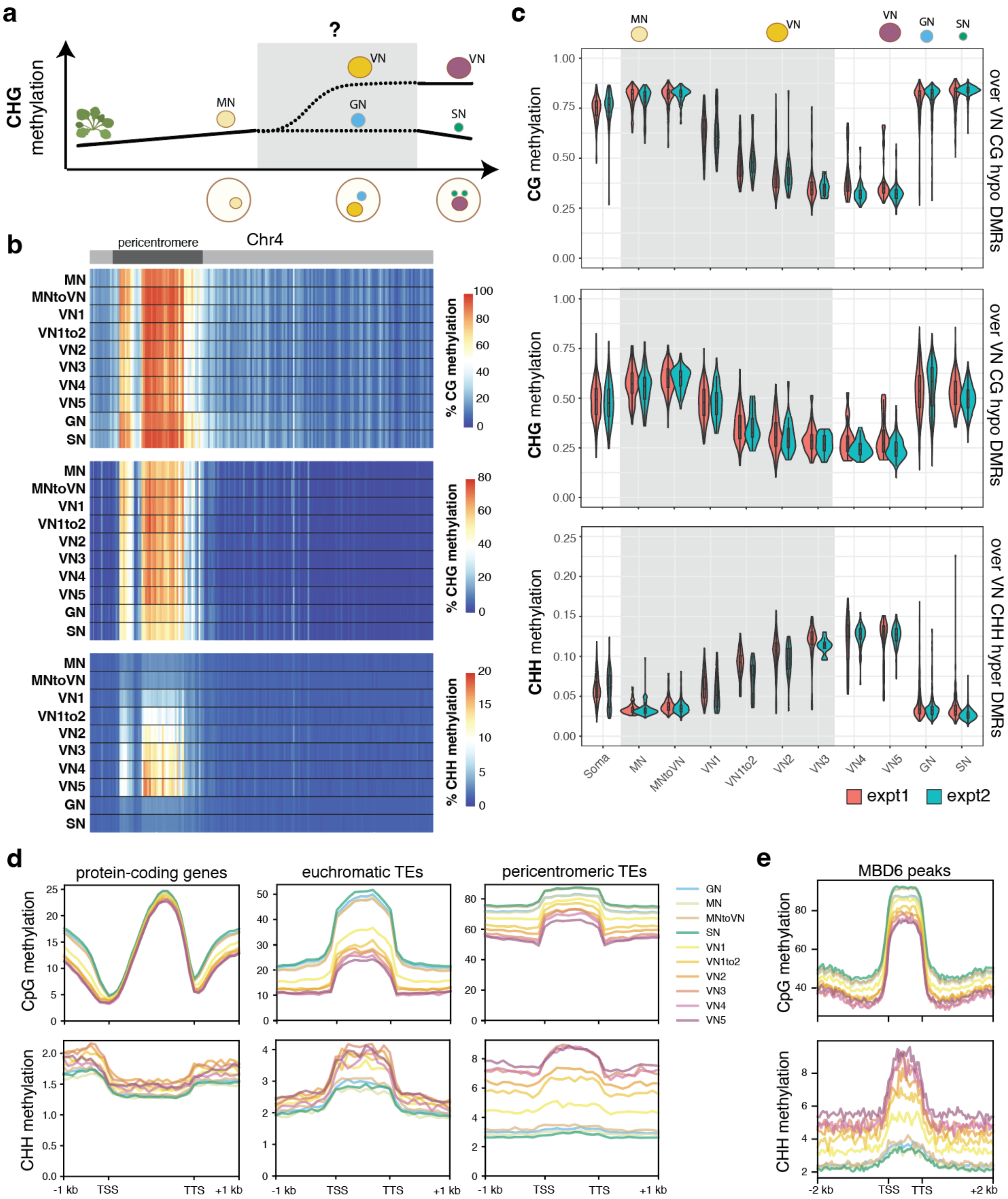

Supplementary Figure 7: Methylation dynamics in Col pollen across snmCT-seq replicates related to Fig. 4. A) Diagram of approximate relative CHG methylation levels during development from vegetative tissue to MN, and finally to mature SN and VN. Rosette image is from BioRender. Picard, C. (2025) https://BioRender.com/vciw129. B) Heatmaps of average CG, CHG and CHH methylation for indicated clusters of nuclei across the length of Chr4, with bins of 100kb. Approximate location of the pericentromere is highlighted in diagram above each plot. C) Distribution of average per-nucleus snmCT-seq methylation

for nuclei assigned to each cluster. Average methylation was computed over regions hypomethylated in the CG context in VN (CG and CHG plots) and hypermethylated in the CHH context in VN (CHH plot), relative to SN. These regions were previously identified from bisulfite-sequencing of purified FACS-sorted VN populations[13]. Nuclei from replicates 1 and 2 shown separately. The number of nuclei used to create the violin plot distributions shown are in Supplementary Data 3 (tab 'nuclei per cluster'). D) Metaplots of methylation data pseudobulked for each cluster indicated, over different genomic annotations. Average of both reps. E) Same as D, but over MBD6 peaks identified from our ChIP-seq.

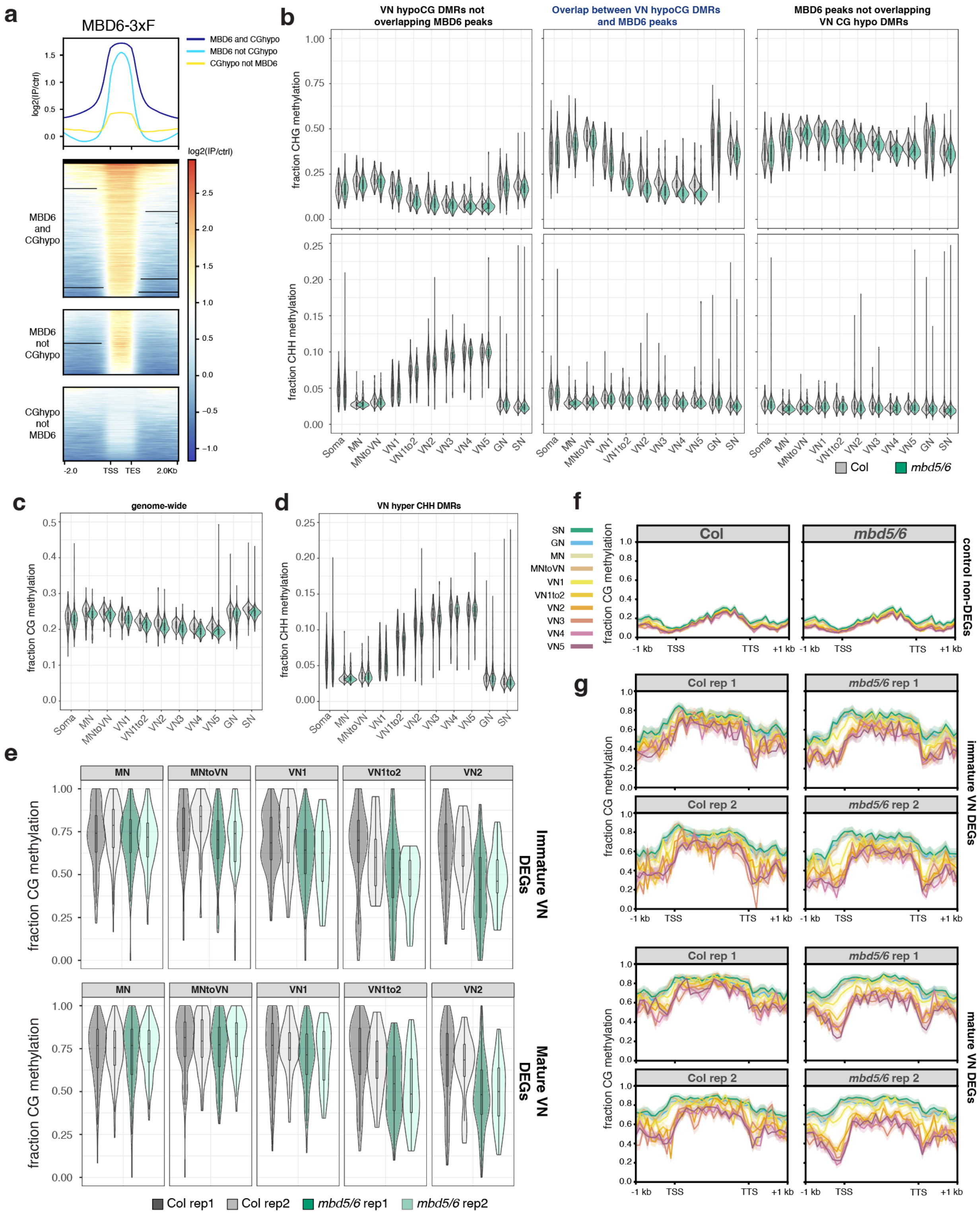

Supplementary Figure 8: Methylation changes in *mbd5/6* pollen across the snmCT-seq dataset related to Fig. 4. (A) MBD6-3xFLAG ChIP-seq signal (avg. of 2 independent lines) in inflorescences over (1) the intersection of MBD6 peaks and VN CG hypo DMRs[13] ("MBD6 and CGhypo"), (2) MBD6 peaks not overlapping VN CG hypo DMRs ("MBD6 not CGhypo"), and (3) VN CG hypo DMRs not overlapping MBD6 peaks ("CGhypo not MBD5/6"). Plotted log2 fold change value is average of two independent transgenic lines. (B) Distribution of snmCT-seq per-nucleus average CHG (top) and CHH (bottom) methylation levels

calculated over the indicated set of regions, comparing Col nuclei to *mbd5/6* nuclei in each pollen nuclei type cluster (x-axis). (C) Distribution of snmCT-seq per-nucleus average CG methylation levels genome-wide, excluding the chloroplast and mitochondrial chromosomes. (D) Distribution of snmCT-seq per-nucleus average CHH methylation levels over VN CHH hyper DMRs[13]. (E) Distribution of snmCT-seq per-nucleus average CG methylation levels over +/-400bp surrounding the TSSs of immature (top) and mature (bottom) *mbd5/6* VN differentially expressed transcripts (Fig. 4e-f) in nuclei of indicated nuclei type cluster and genotype, and replicate, showing both snmCT-seq replicates separately. (F) Metaplots of snmCT-seq methylation profiles pseudobulked across all nuclei in indicated nuclei type cluster, genotype and replicate, for immature and mature VN differentially expressed transcripts (Fig. 4e-f). (B-E) The number of nuclei in each cluster/genotype used to create the violin plot distributions shown are in Supplementary Data 3 (tab 'nuclei per cluster', expt 1 and 2 pooled).

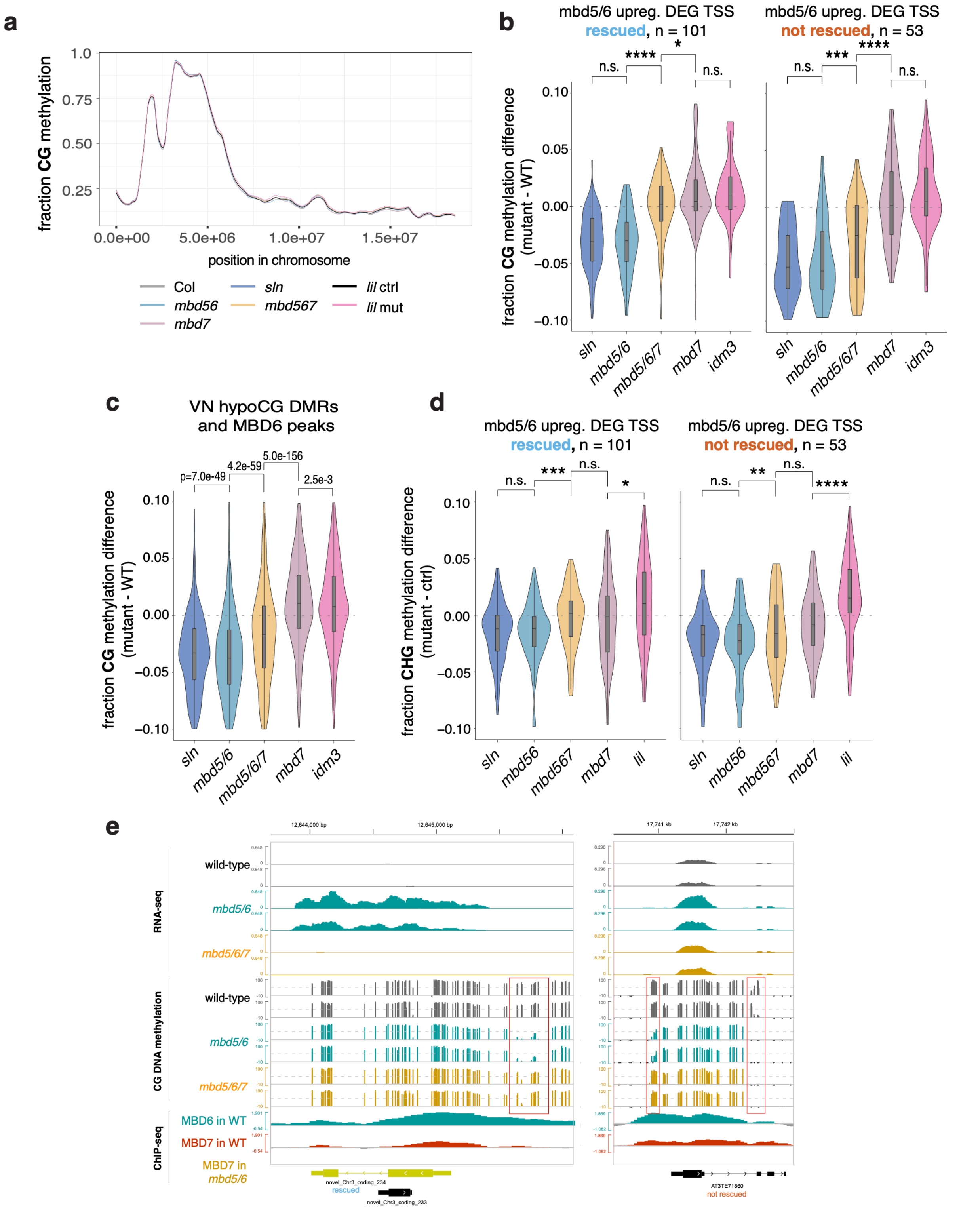

Supplementary Figure 9: Rescue of *mbd5/6* methylation loss in *mbd5/6/7* whole pollen. (A) Smoothed average CG methylation levels across the entire 4th chromosome for whole pollen with indicated genotypes, average of all replicates. (B) Violin plots of average CG methylation difference in indicated mutant vs. wild-type, calculated over the TSS region (+/- 400bp of TSS) of transcriptionally rescued *mbd5/6* upregulated

transcripts (left, n=101) and ‘not rescued’ transcripts (right, n=53). Plotted values are averages across all replicates **** = $p < 0.0001$, *** = $p < 0.001$, ** = $p < 0.01$, * = $p < 0.05$, n.s. = not significant, two-sided paired t-tests. (C) Violin plots of average CG methylation difference in indicated mutant vs. wild-type, calculated over the intersection between VN CG hypo DMRs[13] and MBD6 ChIP-seq peaks. *P*-values are two-sided paired t-tests, number of regions n=7284. (D) Violin plots of average CHG methylation difference in indicated mutant vs. wild-type, calculated over the TSS region (+/- 400bp of TSS) of transcriptionally rescued *mbd5/6* upregulated transcripts (left, n=101) and ‘not rescued’ transcripts (right, n=53). Values are averages across all replicates. Statistical tests and stars representing *p*-values are same as in B. (E) Example genome browser images showing RNA-seq, WGBS, and MBD6 and MBD7 ChIP-seq for a transcriptionally rescued *mbd5/6* upregulated transcript (left) and a ‘not rescued’ transcript. ChIP-seq tracks are average of all independent lines (N=2 for MBD6-Flag, N=3 for MBD7-Flag).

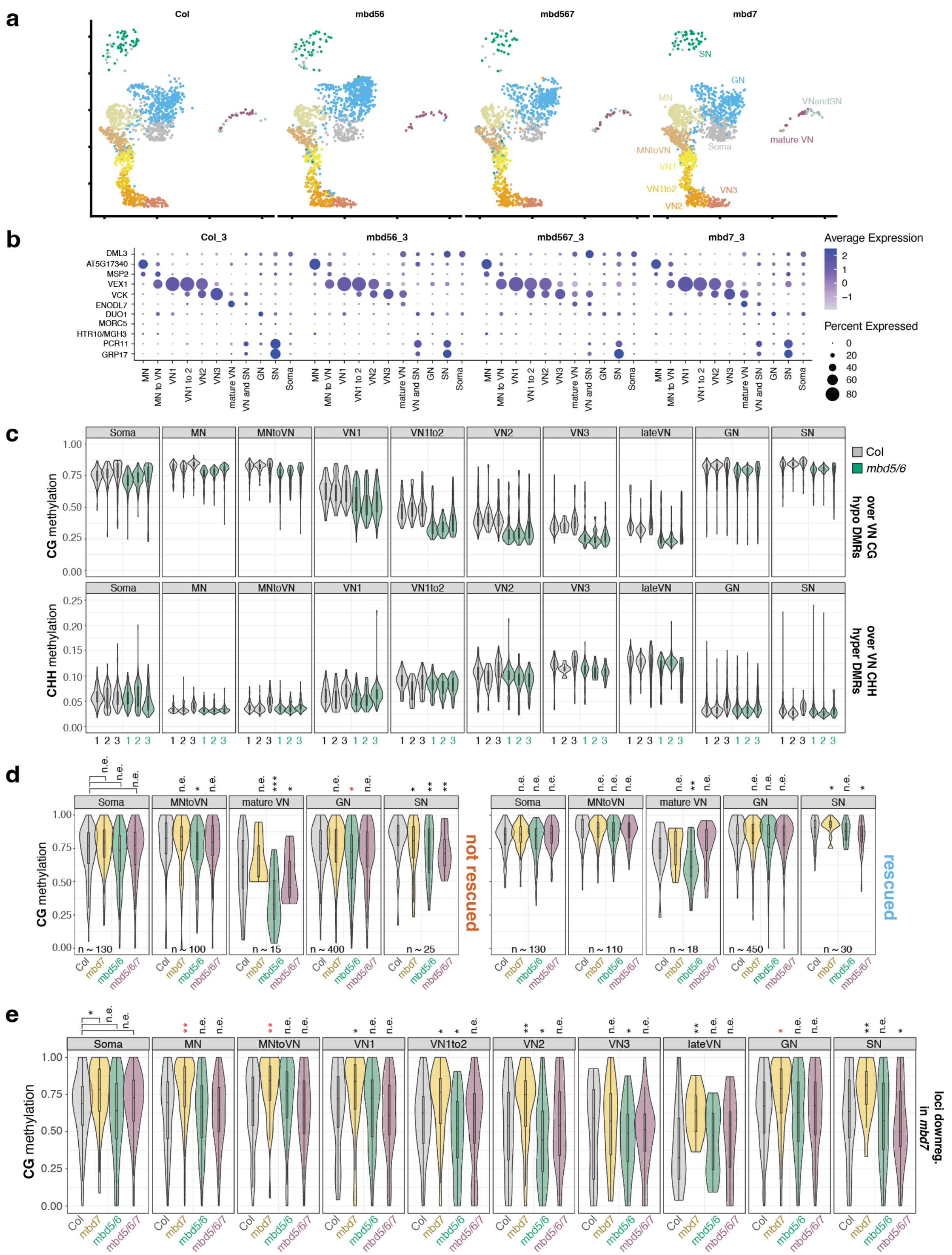

Supplementary Figure 10: Validation of snmCT-seq experiment testing rescue of *mbd5/6* by *mbd7*. (A) UMAPs showing data from each of the four samples from this experiment, projected onto the UMAP from previous experiments 1 + 2 (Fig. 3b). (B) Expression of key marker genes in each cluster (based on projection in A), across each of the four samples in this experiment. DML3 and AT5G17340 are elevated

in MN. The three VN markers are for early post-mitotic (MSP2), mid-stage (VEX1) and late-stage (VCK) VN. ENODL7, DUO1 and MORC5 mark GN, HTR10/MGH3 and PCR11 mark SN, and GRP17 marks somatic nuclei. (C) Comparison of methylation dynamics across all snmCT-seq replicates for Col and *mbd5/6*. CG (top) and CHH (bottom) methylation levels over VN CG hypo DMRs and VN CHH hyper DMRs[13], across nuclei in indicated cluster. Each point in the violin plot represents one nucleus' average methylation over the indicated DMRs. Nuclei from each of the three snmCT-seq experiments (experiments 1 and 2 shown in Fig. 3, experiment 3 in Fig. 5) are shown separately. (D) Average methylation across the four genotypes tested in snmCT-seq experiment 3, across the TSS region of *mbd5/6* upregulated transcripts 'not rescued' (left) or 'rescued' (right) by *mbd7*. Each point in the violin plot represents one nucleus' average methylation over the indicated regions. (E) Same as D, but for loci downregulated in *mbd7* pollen, see Ext. Data Fig. 1e. (D-E) Number of stars indicates Cohen's d. (n.e. = no/minimal effect ($|d| < 0.2$), * = $|d| > 0.2$, ** = $|d| > 0.5$, *** = $|d| > 0.9$, **** = $|d| > 1.5$), red color = $p < 0.001$, two-sided *t*-test. (C-E) The number of nuclei in each cluster/genotype used to create the violin plot distributions shown are in Supplementary Data 3 (tab 'nuclei per cluster').

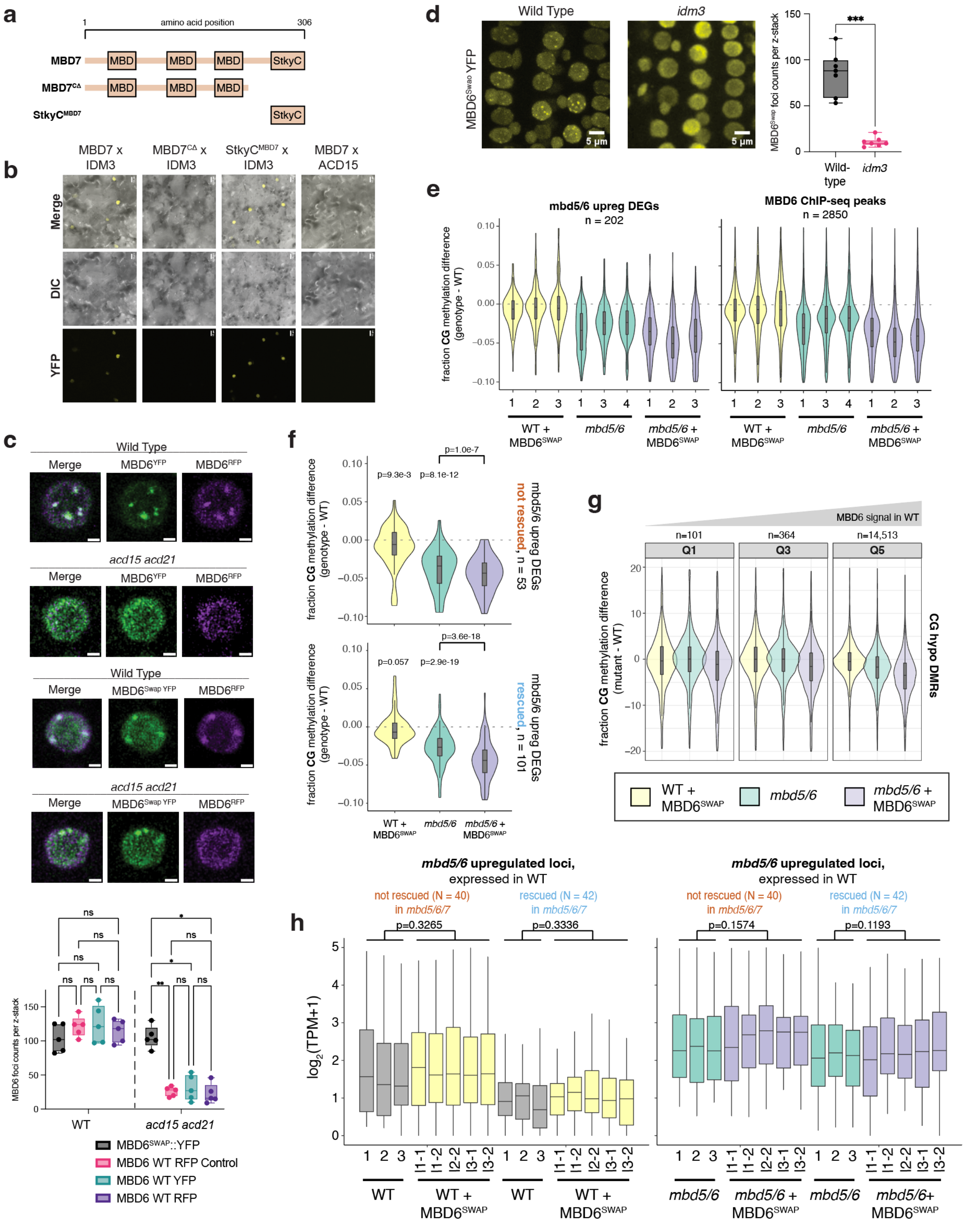

Supplementary Figure 11: Validation of MBD6^{SWAP} related to Fig. 6. (A) Diagram of MBD7 deletions used for BiFC experiments. (B) Example BiFC images testing for interaction between indicated pairs of proteins. (C) Example images (top) and quantification of number of visible foci (bottom) of either wild-type or *acd15 acd21* root nuclei co-expressing a wild-type MBD6 tagged with RFP, and either WT MBD6 or MBD6^{SWAP}

tagged with YFP, from z-stack images of root tips. Significance: 2-way ANOVA with Sidak's multiple comparison test, ** = $p < 0.01$, * = $p < 0.05$, n.s. = $p > 0.05$. (D) YFP fluorescence imaging of root nuclei expressing MBD6^{SWAP}::YFP in either wild-type or *idm3* background. Quantification of foci counts per z-stack at right. Significance: Mann-Whitney test, *** = $p < 0.001$. (E) Distribution of the difference in DNA methylation between wild-type (WT) and indicated genotype, all three replicates per genotype shown. Values were calculated over regions indicated at top of plot. (F) Distribution of the difference in DNA methylation between wild-type (WT) and indicated genotype, average of three replicates. Values were calculated over *mbd5/6* upregulated transcripts whose expression is rescued in *mbd5/6/7* (left, n = 101, see Fig. 1d) or not rescued (n=53, right). Average of three replicates shown. *p*-value = one-sided paired *t*-test. (G) Distribution of the difference in DNA methylation between wild-type (WT) and indicated genotype over 400 bins overlapping VN CG hypo DMRs. The bins were divided into quintiles based on average MBD6 occupancy by ChIP-seq, with Q1 = least MBD6 (n=101), Q3 = middle 20% (n=364), and Q5 = most MBD6 (n=14,513). Note that CG hypo DMRs and MBD6 peaks strongly overlap (Fig. 4a). (H) Distribution of RNA-seq $\log_2$(TPM) for all replicates from Col, Col + MBD6^{SWAP}, *mbd5/6*, and *mbd5/6* + MBD6^{SWAP} (l1, l2, and l3 are different transgenic lines). Values shown are for *mbd5/6* upregulated transcripts that had at least some expression in wild-type, separated based on whether they're rescued (n=42) or not rescued (n=40) in *mbd5/6/7* (Fig. 1d). *p*-value = two-sided paired *t*-test comparing the average of all samples with the same genotype.

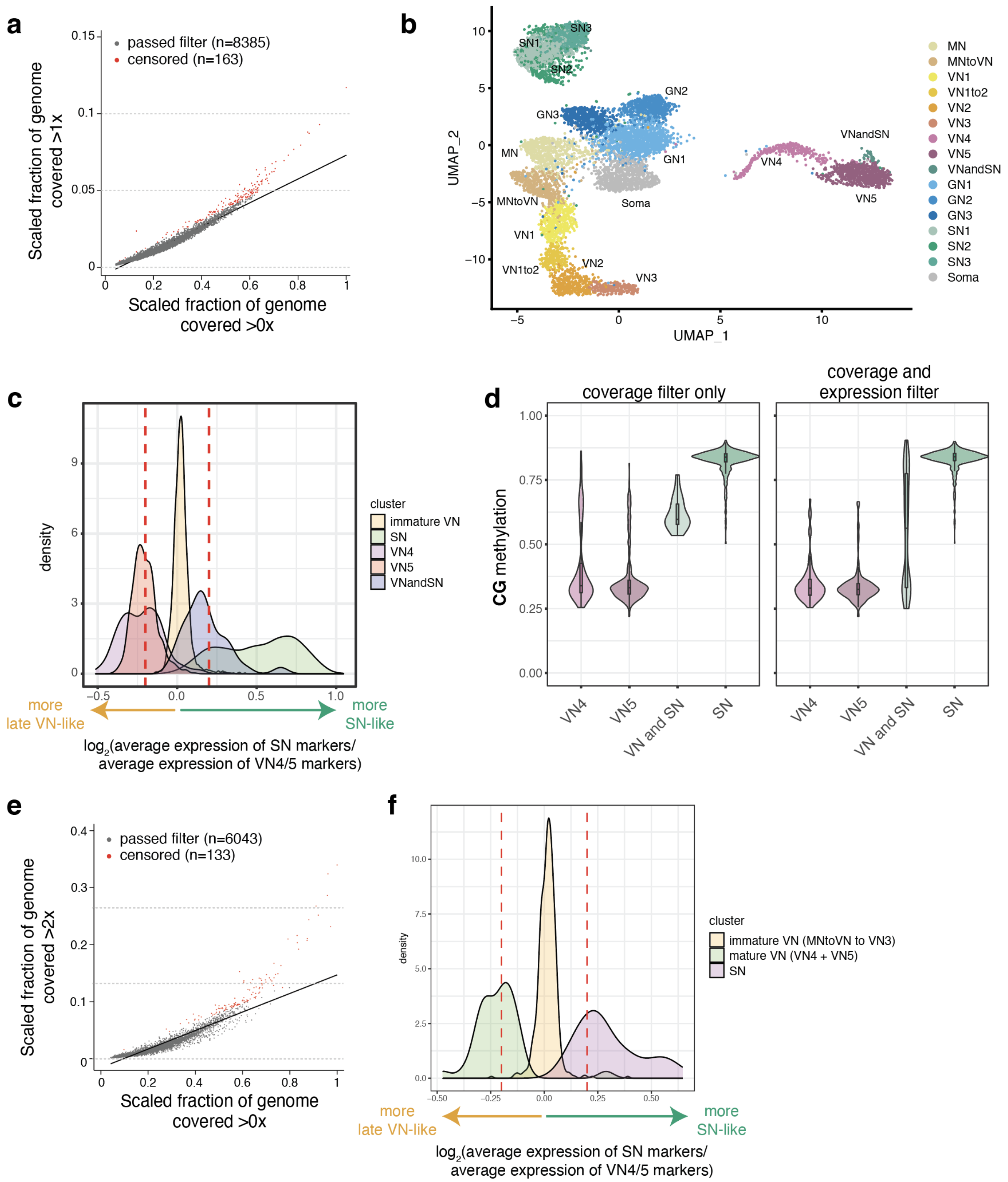

Supplementary Figure 12: Quality control of snmCT-seq dataset – doublet filtering. A) Doublet filtering based on WGBS coverage for snmCT-seq experiments 1+2. Each point represents a single nucleus. x-axis indicates fraction of cytosines in the genome covered by WGBS reads (≥1x), scaled to the nucleus with the highest coverage in the dataset (whose value is set to x = 1). y-axis represents the fraction of the genome covered >1x, using the same scaling. Rates of >1 coverage increase as overall genome coverage increases, but nuclei that showed substantially higher rates of >1 coverage areas were censored as possible doublets (red dots). N = 163 nuclei were censored because of this filter (see methods). B) Initial UMAP plot of all nuclei from snmCT-seq experiments 1+2 passing the filter in (A) and other basic QC filters (see methods), with cluster labels based on marker gene expression. A putative doublet cluster

(‘VNandSN’) has expression of both mature VN and SN markers. C) Doublet filtering based on transcription – experiments 1+2. Log_2 ratio of the average expression of SN markers divided by the expression of mature VN markers for each nucleus, among nuclei in different clusters from (B). Nuclei assigned to the immature VN, as a control, are not enriched for either set of markers. However, VN4, VN5, and SN all have some nuclei falling close to a log_2 ratio of zero (indicating similar expression of mature VN and SN marker genes). Red vertical lines indicate a cutoff of log_2 ratio between -0.2 and 0.2. VN4, VN5, and SN nuclei falling within this region were reassigned to the “VNandSN” cluster as likely doublets. D) Effect of reassigning putative VN/SN doublets identified in (C) to the “VNandSN” cluster in (B). Plot shows distribution of CG methylation levels over VN CG hypo DMRs[13] across all nuclei in indicated cluster, based on different methods to censor doublets. Panel on left shows coverage filter only (see panel A), while right panel shows both coverage filter and the marker expression-based filter in panel C. The number of nuclei in each cluster/genotype used to create the violin plot distributions shown are in Supplementary Data 3 (tab ‘nuclei per cluster’). E) Doublet filtering based on WGBS coverage – experiment 3. F) Doublet filtering based on transcription – experiments 3. VN4 and VN5 were combined into one group (“mature VN”).

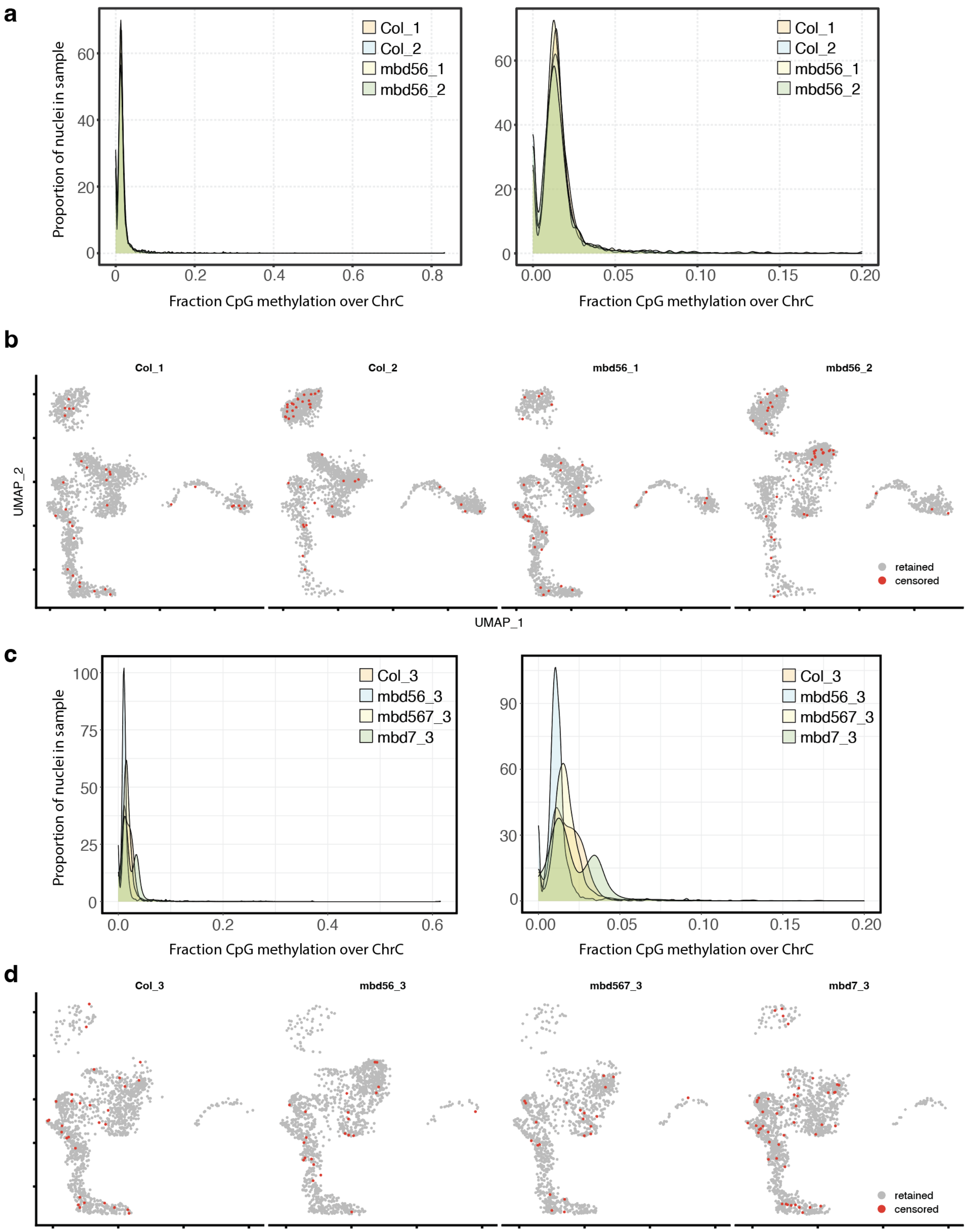

Supplementary Figure 13: WGBS conversion rates in the snmCT-seq data. (A) Average fraction CpG methylation over the chloroplast in each sample in experiments 1 and 2. Right panel is same as left, but zoomed in to x in [0,0.2]. (B) Location of the n = 160 nuclei censored for DNA methylation analysis due to low conversion rates in experiments 1 and 2. (C) Average fraction CpG methylation over the chloroplast in

each sample in experiment 3. Right panel is same as left, but zoomed in to x in [0,0.2]. (D) Location of the n = 124 nuclei censored for DNA methylation analysis due to low conversion rates in experiment 3.

Supplemental References

66. Lorković, Z. J. *et al.* Compartmentalization of DNA Damage Response between Heterochromatin and Euchromatin Is Mediated by Distinct H2A Histone Variants. *Current Biology* **27**, 1192–1199 (2017).

67. Yan, W. *et al.* Dynamic control of enhancer activity drives stage-specific gene expression during flower morphogenesis. *Nat Commun* **10**, (2019).
