# Supplementary material for: An antagonistic epigenetic mechanism regulating gene expression in pollen revealed through single-nucleus multiomics
Source: Nat Commun. 2025 Nov 27;16:10662. doi: 10.1038/s41467-025-65686-z (PMC12660930; doi:10.1038/s41467-025-65686-z)
Supplement: Supplementary file 2 — Description of Additional Supplementary Files [file 41467_2025_65686_MOESM2_ESM.pdf]

Description of Additional Supplementary Files:

Supplementary Data 1: Sequencing data generated for this study. Lists all RNA-seq, snmCT-seq, ChIPseq and WGBS libraries sequenced for this study. Three sets of RNA-seq libraries (lib1-lib3) and five batches of WGBS libraries (lib1-lib5) were sequenced. Col-0 = wild-type, mbd5/6 = mbd5 mbd6 double mutant, mbd7 = mbd7 TDNA (GABI_067_A09) mutant, mbd5/6/7 = mbd5 mbd6 mbd7 triple mutant, MBD6SWAP is a transgene of MBD6 where the MBD6 StkyC has been replaced with the MBD7 StkyC, and line1-3 represent independent transformed lines with the transgene. For snmCT-seq, three separate experiments were performed (expt 1, 2, and 3). For each experiment, Col-0 and mbd5/6 (plus mbd7 and mbd5/6/7 in expt 3) pollen nuclei were sorted into 384 well plates, and wells on a plate were multiplexed and sequenced together. Expt #2 Col plate 8 and mbd5/6 plate 1 failed during library prep and were not sequenced. Pairs with valid barcode = number of read pairs sequenced that had a valid well/nucleus ID barcode, median pairs per well = median read pairs per well for indicated plate, %QC pass = percent of reads obtained for plate that passed basic QC filtering for poor quality reads, % align (RNA) = percent of reads that mapped uniquely to the unconverted transcriptome, % align (WGBS) = percent of reads that did not align to transcriptome and did align to converted genome, % dedup (RNA) = percent of RNA reads that were removed as probable PCR duplicates, % dedup (WGBS) = percent of genomic (bisulfite-converted) reads that were removed as probable PCR duplicates.

Supplementary Data 2: RNA-seq log2(fold change) and TPM estimates. First tab contains the DESeq2 log2(fold change) and adjusted p-value estimates for indicated sample vs. wild-type (Col0) for all lib1 and lib2 samples (see Supplementary Data 1 for list of libraries), as well as for indicated sample + or – SWAP transgene for lib3 samples. The DE analysis of the lib1 and lib2 samples considered all of them together as replicates. Column names are [library pool]_[genotype of log2fc numerator]_v[genotype of log2fc numerator]_[log2fc/padj] where log2fc = log2(fold change) and padj = adjusted p-value. Variables on far right flag significantly DE genes. Second tab contains TPM estimates from Stringtie for all RNA-seq samples. Column names are [library pool]_[genotype]_[replicate]_tpm.

Supplementary Data 3: metadata, quality filtering and cluster assignments for snmCT-seq nuclei. Nuclei are indexed by experiment (1, 2, or 3), genotype (Col-0, mbd5/6, mbd7, mbd5/6/7), plate (1-8) and well (AP1-24). Additionally, each plate has one row indicating

the number of reads from that plate that could not be assigned to any barcode/nucleus. The 'RNA STATISTICS' section gives per-nucleus total reads mapping to the transcriptome, genes detected, and reads not overlapping features, as well as a pass/fail column indicating if the nucleus passed basic filters for RNA-seq data quality (see methods). The 'WGBS COVERAGE' section indicates the percentage of 1,000 bp non-overlapping bins tiled genome-wide that contained at least one cytosine in the indicated sequence context with at least one overlapping WGBS read, as well as a pass/fail column for WGBS data quality (see methods). The 'FINAL ASSIGNMENT' section indicates which nuclei passed the basic initial filtering (pass = both RNA and WGBS portions passed), coverage-based doublet filtering (Supp. Fig. 12a,e), filtering based on elevated mitochondrial/chloroplast RNA reads, overall status (pass/fail) of the nucleus, as well as the cluster ID to which this nucleus was assigned based on Seurat analysis of RNA-seq data. The 'CHLOROPLAST METHYLATION AVERAGES' section indicates, for each nucleus with sufficient quality WGBS data, the average number of sites with data and average methylation in indicated sequence context, only over the chloroplast. Additionally, nuclei censored due to low conversion rates, based on elevated chloroplast methylation, are indicated here. The 'GENOME-WIDE METHYLATION AVERAGES' section is the same as the chloroplast section, but instead over the entire genome excluding the mitochondrial and chloroplast genomes. A second tab lists the number of nuclei assigned to each cluster by experiment and genotype.

Supplementary Data 4: average snmCT-seq methylation data over different genomic regions. For each nucleus, methylation data over all genomic regions in the indicated set of regions was averaged. N_sites = number of covered sites across all regions, avg_cov = average coverage per site, avg_me = average methylation. The cluster assignment of each nucleus is also included.

Supplementary Data 5: image analysis data. Raw data underlying fluorescence quantification, foci counts, and other imaging data shown in this study (specifically Fig. 2c, Fig. 6b, Supp. Fig. 3e-f, and Supp. Fig. 11c-d). Each tab is the data for the indicated figure panel.
