# Supplementary material for: An antagonistic epigenetic mechanism regulating gene expression in pollen revealed through single-nucleus multiomics
Source: Nat Commun. 2025 Nov 27;16:10662. doi: 10.1038/s41467-025-65686-z (PMC12660930; doi:10.1038/s41467-025-65686-z)
Supplement: Supplementary file 8 — Reporting Summary [file 41467_2025_65686_MOESM8_ESM.pdf]

nature portfolio

Corresponding author(s): Steven E Jacobsen

Last updated by author(s): Oct 12, 2025

Reporting Summary

Nature Portfolio wishes to improve the reproducibility of the work that we publish. This form provides structure for consistency and transparency in reporting. For further information on Nature Portfolio policies, see our Editorial Policies and the Editorial Policy Checklist.

Statistics

For all statistical analyses, confirm that the following items are present in the figure legend, table legend, main text, or Methods section.

n/a	Confirmed	
☐	☒	The exact sample size (*n*) for each experimental group/condition, given as a discrete number and unit of measurement
☐	☒	A statement on whether measurements were taken from distinct samples or whether the same sample was measured repeatedly
☐	☒	The statistical test(s) used AND whether they are one- or two-sided<br>*Only common tests should be described solely by name; describe more complex techniques in the Methods section.*
☒	☐	A description of all covariates tested
☐	☒	A description of any assumptions or corrections, such as tests of normality and adjustment for multiple comparisons
☐	☒	A full description of the statistical parameters including central tendency (e.g. means) or other basic estimates (e.g. regression coefficient) AND variation (e.g. standard deviation) or associated estimates of uncertainty (e.g. confidence intervals)
☐	☒	For null hypothesis testing, the test statistic (e.g. *F*, *t*, *r*) with confidence intervals, effect sizes, degrees of freedom and *P* value noted<br>*Give P values as exact values whenever suitable.*
☒	☐	For Bayesian analysis, information on the choice of priors and Markov chain Monte Carlo settings
☒	☐	For hierarchical and complex designs, identification of the appropriate level for tests and full reporting of outcomes
☐	☒	Estimates of effect sizes (e.g. Cohen's *d*, Pearson's *r*), indicating how they were calculated

Our web collection on statistics for biologists contains articles on many of the points above.

Software and code

Policy information about availability of computer code

Data collection	none
Data analysis	All published software used to analyze the data in this study are reported in the methods section. All custom code developed to analyze these data are available on github at: https://github.com/clp90/MBD_antagonism.

For manuscripts utilizing custom algorithms or software that are central to the research but not yet described in published literature, software must be made available to editors and reviewers. We strongly encourage code deposition in a community repository (e.g. GitHub). See the Nature Portfolio guidelines for submitting code & software for further information.

Data

Policy information about availability of data

All manuscripts must include a data availability statement. This statement should provide the following information, where applicable:

- Accession codes, unique identifiers, or web links for publicly available datasets
- A description of any restrictions on data availability
- For clinical datasets or third party data, please ensure that the statement adheres to our policy

The sequencing data generated in this study have been deposited in the Gene Expression Omnibus (GEO) database under accession code GSE275832 [https://www.ncbi.nlm.nih.gov/geo/query/acc.cgi?acc=GSE275832]. Published data used in this study: MBD5-Myc and MBD6-Myc (GSM5267036 [https://www.ncbi.nlm.nih.gov/geo/query/acc.cgi?acc=GSM5267036], GSM5267037 [https://www.ncbi.nlm.nih.gov/geo/query/acc.cgi?acc=GSM5267037], GSM5267038 [https://www.ncbi.nlm.nih.gov/geo/query/acc.cgi?acc=GSM5267038])4, H3K9me2 (GSM3130575 [https://www.ncbi.nlm.nih.gov/geo/query/acc.cgi?

acc=GSM3130575], GSM3130576 [https://www.ncbi.nlm.nih.gov/geo/query/acc.cgi?acc=GSM3130576])60, NRPE1 (GSM2667838 [https://www.ncbi.nlm.nih.gov/geo/query/acc.cgi?acc=GSM2667838], GSM2667837 [https://www.ncbi.nlm.nih.gov/geo/query/acc.cgi?acc=GSM2667837])43, 10x snRNA-seq of pollen nuclei (GSE202422 [https://www.ncbi.nlm.nih.gov/geo/query/acc.cgi?acc=GSE202422])6. The raw imaging data generated in this study are available in the Dryad database [https://doi.org/10.5061/dryad.vx0k6dk23]. Seurat objects for the snmCT-seq data analysis are available on github at: https://github.com/clp90/MBD_antagonism. All additional data used to generate figures are provided in Supplementary Data 1-5.

Research involving human participants, their data, or biological material

Policy information about studies with human participants or human data. See also policy information about sex, gender (identity/presentation), and sexual orientation and race, ethnicity and racism.

Reporting on sex and gender	n/a
Reporting on race, ethnicity, or other socially relevant groupings	n/a
Population characteristics	n/a
Recruitment	n/a
Ethics oversight	n/a

Note that full information on the approval of the study protocol must also be provided in the manuscript.

Field-specific reporting

Please select the one below that is the best fit for your research. If you are not sure, read the appropriate sections before making your selection.

☒ Life sciences ☐ Behavioural & social sciences ☐ Ecological, evolutionary & environmental sciences

For a reference copy of the document with all sections, see nature.com/documents/nr-reporting-summary-flat.pdf

Life sciences study design

All studies must disclose on these points even when the disclosure is negative.

Sample size	All RNA-seq was performed at least in triplicate. All ChIP-seq and WGBS was performed in duplicate. For experiments using transgenic lines, at least two independent lines were used. Single-cell experiments were performed in triplicate for wild-type and mbd5/6, with the last replicate also including mbd7 and mbd5/6/7 For imaging studies, number of nuclei assessed is indicated in each plot. In most cases, multiple nuclei were assessed from the same root image.
Data exclusions	none
Replication	Key RNA-seq experiments were performed twice (3 reps each) with very consistent results. Single-nucleus RNA + methylome data generation + analysis was performed in three independent experiments, again highly consistent although with some difference in the staging/age of the plants affecting the ratio of immature to mature pollen nuclei. ChIP-seq was performed with multiple independent lines with similar protein expression levels.
Randomization	n/a
Blinding	n/a

Reporting for specific materials, systems and methods

We require information from authors about some types of materials, experimental systems and methods used in many studies. Here, indicate whether each material, system or method listed is relevant to your study. If you are not sure if a list item applies to your research, read the appropriate section before selecting a response.

Materials & experimental systems

n/a	Involved in the study	
☐	☒	Antibodies
☒	☐	Eukaryotic cell lines
☒	☐	Palaeontology and archaeology
☒	☐	Animals and other organisms
☒	☐	Clinical data
☒	☐	Dual use research of concern
☐	☒	Plants

Methods

n/a	Involved in the study	
☐	☒	ChIP-seq
☐	☒	Flow cytometry
☒	☐	MRI-based neuroimaging

Antibodies

Antibodies used	Anti-FLAG antibody (Sigma) was used at a 1:800 dilution for ChIP-seq
Validation	Our lab has used this antibody extensively. We performed this ChIP-seq for MBD7 alongside MBD6, which we have already published in earlier work, and the MBD6 data was highly consistent with our previous results. Additionally, the no-FLAG controls confirm no non-specific binding of the antibody.

Dual use research of concern

Policy information about dual use research of concern

Hazards

Could the accidental, deliberate or reckless misuse of agents or technologies generated in the work, or the application of information presented in the manuscript, pose a threat to:

No	Yes	
☒	☐	Public health
☒	☐	National security
☒	☐	Crops and/or livestock
☒	☐	Ecosystems
☒	☐	Any other significant area

Experiments of concern

Does the work involve any of these experiments of concern:

No	Yes	
☒	☐	Demonstrate how to render a vaccine ineffective
☒	☐	Confer resistance to therapeutically useful antibiotics or antiviral agents
☒	☐	Enhance the virulence of a pathogen or render a nonpathogen virulent
☒	☐	Increase transmissibility of a pathogen
☒	☐	Alter the host range of a pathogen
☒	☐	Enable evasion of diagnostic/detection modalities
☒	☐	Enable the weaponization of a biological agent or toxin
☒	☐	Any other potentially harmful combination of experiments and agents

Plants

Seed stocks	All plants used were Arabidopsis ecotype Col-0. The following TDNA mutant lines were obtained from Arabidopsis Biological Resource Center (ABRC): mbd7-3 (GABI_067_A09), sln (SALK_090484), mbd6-1 (SALK_043927). The mbd5/6 double mutant used in this study is the previously described mbd5/6 CRISPR. The idm3/lil-1 and YJ control seeds were kindly donated by the Law lab.
Novel plant genotypes	The mbd5 mbd6 mbd7 triple mutant was generated using the pYAO::hSpCas9 system37 to edit MBD5 and MBD7 in the mbd6-1 (SALK_043927) genetic background. Briefly, we cloned sequentially a guide for the MBD5 gene (ACCGGAGAACCCGGCTACTC) and a guide for the MBD7 gene (GCTATTCACAGACACTTGGC) into the pYAO::hSpCas9 plasmid by overlapping PCR. The plasmid was transformed into Agl0 agrobacteria, which were used to transform mbd6-1 plants via floral dipping. Mutations were screened in T1 transgenic lines by Sanger sequencing. The plants used in this study are T2 or T3 Cas9 negative segregants, with homozygous mutations.
Authentication	All TDNA lines were validated using the standard PCR approach. All CRISPR lines were validated by sequencing.

ChIP-seq

Data deposition

☒ Confirm that both raw and final processed data have been deposited in a public database such as GEO.

☒ Confirm that you have deposited or provided access to graph files (e.g. BED files) for the called peaks.

Data access links<br>*May remain private before publication.*	These are in a private github repository, will be made public on publication

Files in database submission	GEO repository contains raw data and bigwig tracks.
Genome browser session (e.g. UCSC)	Bigwig files are included in the GEO repository.

Methodology

Replicates	Two replicates per genotype, and two independent transgenic lines.
Sequencing depth	Approximately 60-100 million reads per sample aligned uniquely and remained after removing PCR duplicates.
Antibodies	For ChIP-seq: anti-FLAG antibody (Sigma, Monoclonal ANTI-FLAG M2, cat. no. F1804) was used at a 1:800 dilution<br>For Western Blots: anti-FLAG-HRP antibody (Sigma, Monoclonal ANTI-FLAG M2-Peroxidase, cat. no. A8592) at 1:10,000 dilution
Peak calling parameters	Peaks were identified using MACS2 v3.0.0a7 callpeak with options -g 119000000 --seed 123456 --broad -f BAMPE --broad-cutoff 0.1. Peaks from replicates were combined using bedtools merge.
Data quality	While there was variation in signal/noise ratio (presumably due to different levels of expression of the transgenes), the pattern of enrichment over different types of peaks (Fig. 2A) was highly consistent.
Software	MACS2 v3.0.0a7; Deeptools v3.0.2 and v3.5.5 ; bowtie2 v2.3.4.3; TrimGalore v0.6.7; STAR v2.7.9a; Picard Tools' MarkDuplicates v1.121; DESeq2 v1.34.0; StringTie v2.1.6; Bismark v2.1.6; R v4.4.0; ggplot2 v.3.5.1; Seurat v.4.0.4; pheatmap v1.0.12;

Flow Cytometry

Plots

Confirm that:

- [ ] The axis labels state the marker and fluorochrome used (e.g. CD4-FITC).
- [ ] The axis scales are clearly visible. Include numbers along axes only for bottom left plot of group (a 'group' is an analysis of identical markers).
- [ ] All plots are contour plots with outliers or pseudocolor plots.
- [ ] A numerical value for number of cells or percentage (with statistics) is provided.

Methodology

Sample preparation	Sample preparation for snmCT-seq was performed as previously described for snRNA-seq with the 10X genomics platform, with a minor variation in the last step prior to nuclei sorting6. Briefly, mixed spores were isolated from open flowers, and pollen was broken to release nuclei into solution. Nuclei were then filtered and collected by centrifugation. Nuclei pellets were resuspended in 2 mL nuclei extraction buffer and DAPI buffer combined in a 1:8 ratio (Cystain UV Precise P, Sysmex). The Col and mbd5/6 samples were then split into two aliquots, which were sorted simultaneously on two machines.
Instrument	a BD FACS ARIAII instrument and BD FACS ARIA
Software	FACSDiva
Cell population abundance	FACS was used to separate nuclei (based on size + DAPI intensity) from debris and sort them individually into a 384 well plate, no additional sorting/filtering was performed
Gating strategy	As previously described in Ichino et al. 2022 Cell Reports (gating strategy is shown in Fig. S5A-S5C).

- [ ] Tick this box to confirm that a figure exemplifying the gating strategy is provided in the Supplementary Information.
